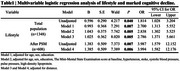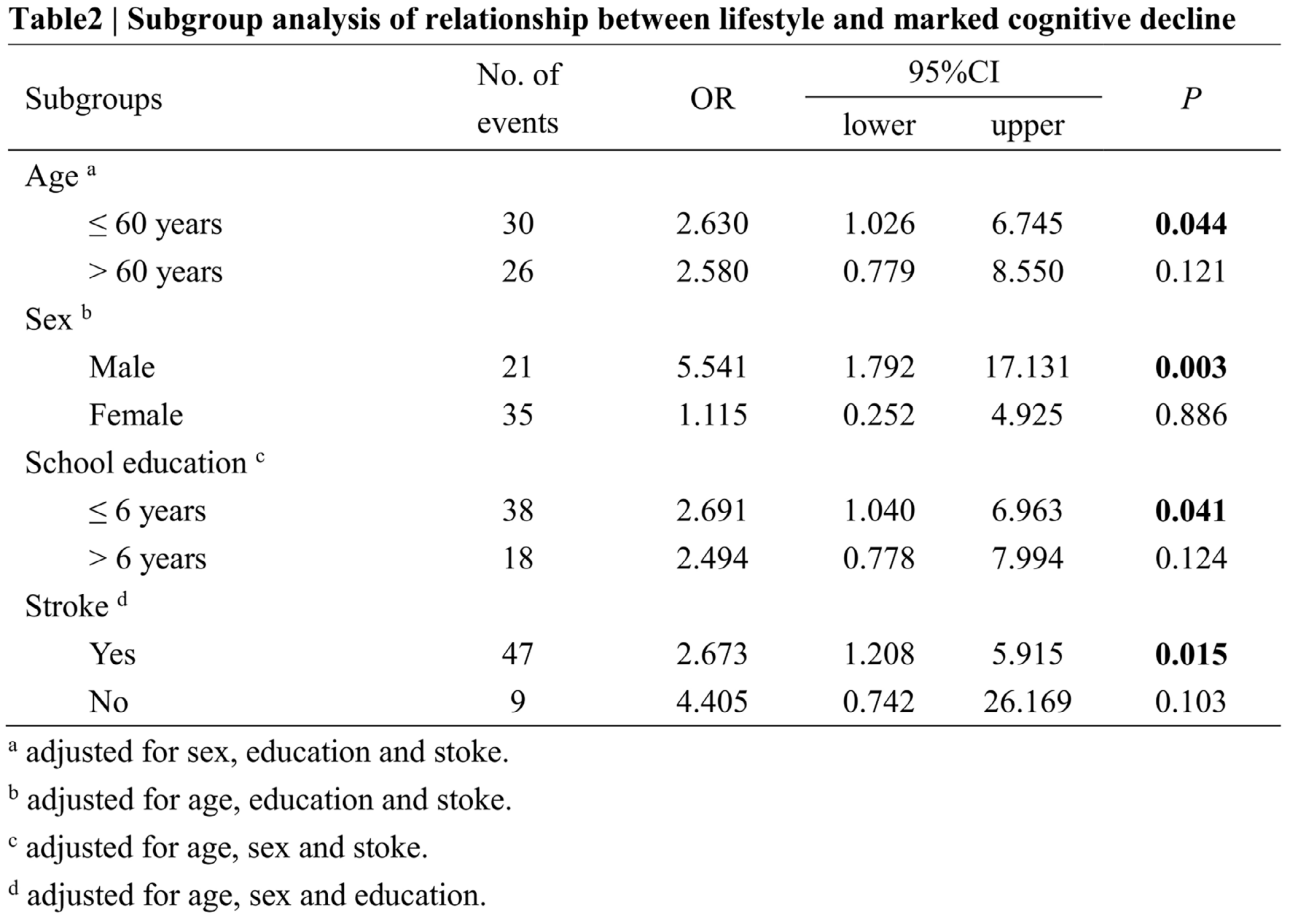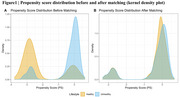# The relationship between lifestyle and cognitive decline among people aged 40 years and older: A community‐based 4‐year prospective cohort study

**DOI:** 10.1002/alz70860_101493

**Published:** 2025-12-23

**Authors:** Rong Zhou, Suhang Shang, Ling Gao, Liangjun Dang, Shan Wei, Chen Chen, Jingyi Wang, Jin Wang, Qiumin Qu, Yan Qu

**Affiliations:** ^1^ The First Affiliated Hospital of Xi'an Jiaotong University, Xi'an, Shaanxi, China; ^2^ Huxian Hospital of Traditional Chinese Medicine, Xi'an, Shaanxi, China

## Abstract

**Background:**

Several unhealthy lifestyles have been identified as potential risk factors for cognitive impairment, however the effect of combinations of lifestyles are ambiguous. In the present study, we examined the association of lifestyles with cognitive decline among cognitively normal people aged 40 years and older.

**Method:**

This was a communicate population based cohort study, using a cluster random sampling to select a population of 2 villages in Xi'an, and cognitively normal subjects were followed up for 4 years. A comprehensive score of lifestyle was calculated based on the factors including smoking, drinking, exercise, and diet collected at the baseline. The Mini‐Mental State Examination (MMSE) was used to evaluate global cognitive function at both baseline and follow‐up, and a drop of ≥ 4 points in MMSE score from baseline was defined as significant cognitive decline. Multivariable logistic regression, propensity score correction and propensity score matching were used to investigate the relationship between lifestyle and cognitive decline.

**Result:**

1348 participants were ultimately enrolled and 56 (4.2%) met the criteria for significant cognitive decline (ΔMMSE ≥ 4 points). 304 (22.6%) met the definition of the unhealthy lifestyle (comprehensive score <6). Multivariable logistic regression analysis showed that unhealthy lifestyle was positively associated with significant cognitive decline (OR=2.780, 95% CI 1.345‐5.748, *p* = 0.006). Propensity‐score adjusted model yielded similar result (OR=2.786, 95% CI 1.371‐5.661, *p* = 0.005) (Table 1). Propensity score matching was performed to balance the differences in covariates between the two groups (Figure 1). Multivariate logistic regression analysis conducted in the matched population revealed the risk of significant cognitive decline was still higher for those with unhealthy lifestyle (OR=3.994, 95% CI 1.582‐12.176, *p* = 0.006). Stratified analysis found an association between lifestyle and cognitive decline in participants who were aged ≤60 years (OR=2.630, 95% CI 1.026‐6.745, *p* = 0.006), male (OR=5.541, 95% CI 1.792‐17.131, *p* = 0.003), and who had school education ≤6 years (OR=2.691, 95% CI 1.040‐6.963, *p* = 0.041) or stroke history (OR=2.673, 95% CI 1.208‐5.915, *p* = 0.015) (Table 2).

**Conclusion:**

Unhealthy lifestyle is associated with an increased risk of cognitive decline in people aged 40 years and older, particularly in the male, middle‐aged, low‐educated and with a history of stroke.